# Perceptions of community-based field workers on the effect of a longitudinal biomedical research project on their sustainable livelihoods

**DOI:** 10.1186/s12889-017-4138-6

**Published:** 2017-03-17

**Authors:** Christabelle S. Moyo, Joseph Francis, Pascal O. Bessong

**Affiliations:** 1 HIV/AIDS & Global Health Research Programme, School of Mathematical and Natural Sciences, University of Venda, Private Bag X5050, Thohoyandou, 0950 South Africa; 2 Institute for Rural Development, School of Agriculture, University of Venda, Private Bag X5050, Thohoyandou, 0950 South Africa

**Keywords:** MAL-ED, Biomedical research, Community-based field workers, Perceptions, Sustainable livelihoods, South Africa

## Abstract

**Background:**

Researchers involved in biomedical community-based projects rarely seek the perspectives of community fieldworkers, who are the ‘foot soldiers’ in such projects. Understanding the effect of biomedical research on community-based field workers could identify benefits and shortfalls that may be crucial to the success of community-based studies. The present study explored the perceptions of community-based field workers on the effect of the Etiology, Risk Factors and Interactions of Enteric Infections and Malnutrition and the Consequences for Child Health and Development Project" (MAL-ED) South Africa on their tangible and intangible capital which together comprise sustainable livelihoods.

**Methods:**

The study was conducted in Dzimauli community in Limpopo Province of South Africa between January-February 2016. The sustainable livelihoods framework was used to query community-based field workers’ perspectives of both tangible assets such as income and physical assets and intangible assets such as social capital, confidence, and skills. Data were collected through twenty one individual in-depth interviews and one focus group discussion. Data were analysed using the Thematic Content Analysis approach supported by ATLAS.ti, version 7.5.10 software.

**Results:**

All the field workers indicated that they benefitted from the MAL-ED South Africa project. The benefits included intangible assets such as acquisition of knowledge and skills, stronger social capital and personal development. Additionally, all indicated that MAL-ED South Africa provided them with the tangible assets of increased income and physical assets. Observations obtained from the focus group discussion and the community-based leaders concurred with the findings from the in-depth interviews. Additionally, some field workers expressed the desire for training in public relations, communication, problem solving and confidence building.

**Conclusions:**

The MAL-ED South Africa, biomedical research project, had positive effects on tangible and intangible assets that compose the sustainable livelihoods of community-based fieldworkers. However, the field workers expressed the need to acquire social skills to enable them carry out their duties more efficiently.

## Background

Exploring the views of various interest groups involved in community-based projects is an integral component of community-based participatory research, yet it is rarely part of biomedical scientific research projects. Biomedical researchers involved in community-based projects mainly concentrate on advancing medical knowledge, improving treatment, care and quality of life [1] and generally pay minimal attention to the social aspects of their work. Although studies have investigated the perceptions of research participants and what drives them to be involved in biomedical research projects [2–6], there is little information on the effect of participating in biomedical studies on field workers, who are the real ‘foot soldiers’ or frontline workers. Input from field workers could inform decisions about how to train workers to ensure study quality, and could aid in planning and implementing future research initiatives. This kind of investigation would also reveal the effects of community-based research beyond those of generating new knowledge. The foregoing issues led to this study which explored the views of community-based field workers on the effects of working on the Etiology, Risk Factors and Interactions of Enteric Infections and Malnutrition and the Consequences for Child Health and Development Project" (MAL-ED) South Africa on their tangible and intangible capital that compose sustainable livelihoods.

The Sustainable Livelihoods Framework of the Department for International Development (DfID) of the United Kingdom provided a way to view potential effects of the MAL-ED South Africa study on field workers. This framework outlines human, financial, physical, social and natural capitals as five key assets and capabilities that help people to sustain their own livelihoods [7]. According to the framework, human capital encompasses aspects such as skills, knowledge, good health and physical capabilities which enable people to undertake various initiatives in order to improve their livelihoods. Financial or economic capital includes cash, savings, credit, economic assets, remittances of all types which serve as ‘catalysts’ for realising sustainable livelihoods. Shelter, household goods and productive equipment constitute physical capital. Moreover, social capital is embedded within the framework of creating friendships, family support, partnerships and networks. Lastly, water, soil, air and other environmental sources from which human life derives its existence make up natural capital. Scholars such as [8] have added personal assets as another crucial dimension that supplements or complements the sustainable livelihoods framework. Personal assets include self- esteem, self-confidence, motivation and emotional well-being, all of which are positive attributes of sustainable living.

Some researchers and institutions view the knowledge that grassroots community members possess as of insignificant value [9]. This view overlooks the potential to access and utilise grassroots knowledge to enhance the development, implementation and evaluation of research initiatives. Community-based field workers play a pivotal role in research projects, devoting time and energy to collecting data in understudied areas [10]. They determine to a large extent the success or failure of a project or programme. The data they collect should be of good quality and credible [11]. Understanding the opinions and perspectives of the field workers could provide a more balanced understanding of the range of effects of community-based projects. Community participation may allow for the sharing of accurate information between researchers and the participating communities [1]. This exchange of information could serve as a valuable empowerment tool for both researchers and project participants [11]. Furthermore, exchanging ideas precipitates a sense of belonging for the study participants [1, 2] if they see themselves as active participants and joint owners of the research process.

Investigating the perceptions of community-based field workers when evaluating a scientific biomedical study such as the MAL-ED project might result in a more holistic understanding of community-based research and highlight its relevance to community members. When community members understand the purpose of the study and its possible effects on their livelihoods they often become eager and willing to participate. Thus, researchers should make concerted efforts to actively involve persons who play a central role in community-based projects or programmes.

The current study focused on the MAL-ED South Africa collaborative project, which was initiated in 2009, involving the University of Venda, South Africa and the University of Virginia, United States of America. It was an observational, prospective, community-based birth cohort study conducted in the Dzimauli community of Limpopo Province in South Africa. The study was designed to investigate the interactions of enteropathogens, malnutrition, growth and cognitive development in young children. Field workers were recruited from the community and trained to collect data on childhood illnesses, vaccination history, feeding habits; and to collect biospecimens such as stool and urine, following standard protocols governing the eight field sites of the MAL-ED network [12].

This paper is a product of engaging the community-based field workers so that they could share their views on the effects of the MAL-ED South Africa project on their sustainable livelihoods. In this paper the term ‘livelihoods’ encompasses all factors that assist in developing and enhancing the capabilities of community-based fieldworkers to enable them to enjoy basic necessities of life, including tangible assets such as income and physical assets and intangible assets such as skills, knowledge, social factors and personal development.

Although community leaders were not directly involved in the study, their views were also solicited in order to find out the effects of the MAL-ED project on the livelihoods of community members under their care. Thus, the views of the community leaders were sought to corroborate the findings of the in-depth individual field workers’ interviews.

## Methods

### Study site

As already indicated above, the study was conducted in Dzimauli community in Mutale Municipality between January and February 2016. The geographical positioning coordinates of Mutale Municipality are 22°44′13″S 30°31′34″E. It is located approximately 200 km to the north of Polokwane, the capital city of Limpopo Province in northern South Africa. The demographic, socio-economic and health indicators of the community have been described [12]. Briefly, Dzimauli community is made up of nine villages, namely Bhayimora, Madadani, Mbayela, Pile, Tshandama, Tshangwa, Thongwe, Tshapasha, and Tshibvumo. The community has a population of about 9 000 inhabitants of black Africans, with a female proportion of 53%. The population is of low socio-economic status, with high levels of malnutrition [13, 14].

### Research design and approach

A Case study design was employed in this current study. A qualitative approach was used to explore the views of the study participants regarding the effects of the MAL-ED South Africa project on their livelihoods. A qualitative approach was considered appropriate for this study because it provides a better understanding of the phenomenon through directly and deeply engaging participants. Qualitative research approaches are commonly used to explore, interpret or obtain a ‘deeper understanding’ of certain aspects of human beliefs, attitudes or behavior [15]. The purposive sampling technique, also known as judgmental sampling, was used to select study participants. Purposive sampling was considered ideal for this study because all the community-based field workers who had taken part in the MAL-ED South Africa project were eligible to participate in the current study. Community leaders though not directly involved in the MAL-ED project were also purposively selected by virtue of their being custodians of their communities on issues such as tradition, culture, values, land and other day to day to day activities. Furthermore, some of them assisted in the recruitment of field workers from the community.

### Study population

As alluded to in the previous section, all the 22 community-based field workers who had participated in the initial phase of the MAL-ED South Africa project (24 months of study subject follow up) and nine Dzimauli community leaders constituted the target population for the current study. Four of the 22 based field workers could not be reached to be consented personally or by phone; they had relocated to other provinces in search of jobs. Two field workers declined to participate. They did not give reasons as to why they were unwilling to participate in the study. Attempts to obtain reasons for refusal were unsuccessful. However, it was realized that the two individuals who declined to participate has been let go of their duties due to unsatisfactory performance, and this could have been the reason for their refusal. Therefore, 16 field workers consented to participate in the study. Out of the 9 community leaders, four were unavailable for the individual interviews due to other competing engagements. With no forthcoming proposed timeframes from them to participate, it was considered unreasonable to continue to wait without basis. Therefore, five community leaders were consented to participate in the study.

### Data collection and analysis

In depth interview guides and focus group discussion were chosen as the data collection methods to ensure that there was triangulation of methods of data collection [16]. Triangulation of methods was applied to corroborate and ensure the credibility of the findings from the in-depth individual interviews. The questions on the interview guides were checked by other investigators, not associated with the current study, who have the relevant expertise and knowledge in the area to ensure that the questions were correctly phrased and that they captured all the information needed by the study. Furthermore, pilot testing the research instruments made it possible to test the reliability of the research instruments. Added to that, the use of an audio tape recorder made it possible to accurately capture the participants’ views.

Face to face individual interviews were conducted with 12 community-based field workers. Four community-based field workers who were unable to schedule face to face sessions were interviewed telephonically. Five community-based leaders were also interviewed in order to find out their perspectives on the effects of the project on the livelihoods of field workers residing in the community. The interview guide used was in English and Tshivenda (the local language of the community). Both the individual and focus group discussion were conducted in English. The services of an interpreter were only sought when participants preferred to use Tshivenda instead of English.

The interview guide for the community-based field workers contained the following questions:

- What are your general views about the MAL-ED South Africa project?
- What knowledge and skills can you recall having prior to joining the MAL-ED project?
- What knowledge and skills do you believe you gained as a result of your involvement in MAL-ED project?
- How have the knowledge and skills you acquired through your involvement in MAL-ED project helped you?
- What other skills could have helped you execute your duties more effectively in the MAL-ED project?
- What other benefits (if any) did you get as a result of your participation in the MAL-ED project?
- What did you dislike about the project?
- If the project was to be repeated, what things would you want changed?
- If a project of this nature was to be carried out again, would you be willing to take part in it? If yes, why?
- Would you advise people to take part in the project if it were to be repeated in your community? Give reasons for your answer.
- Has taking part in the MAL-ED project influenced your general understanding of research? If so how?
- Has your involvement in the MAL-ED project influenced your career prospects/career? If so, in what way?

The community leaders’ interview guide consisted of 6 relevant questions selected from the community-based field workers’ interview guide. The questions included their general view of the study; benefits of the study to community-based field workers; what they thought the community-based field workers did not like about the project; what they thought the community-based field workers would like to see changed; whether they thought the community-based field workers would be willing to participate in future studies of this nature; and if they would encourage community members to participate as field workers in future projects of this nature.

### Piloting of the interview guide

The research instruments were pilot-tested with 4 participants. This was done in order to gauge the time taken to conduct each interview, clarity of the questions and ease of understanding the questions. The participants were given a chance to express their opinions regarding the interview guide, whether the questions were clear, other issues that they wanted captured in the interview guide and whether enough time was allocated for the interview. After the pilot testing exercise, the research instruments were adjusted. Questions that required similar information were grouped together and those that required clarity were rephrased using simpler language. Also, the time taken for the interviews was adjusted accordingly.

In-depth interviews were conducted first with the community-based field workers and then the community leaders. Community leaders were only involved in face to face interviews and not in focus group discussion because they were not the focus of the study but were there to corroborate the findings of the community-based field workers. Participants were made aware of the need for both parties (interviewer and interviewee) to maintain confidentiality regarding the information shared during the interviews. The purpose of the in-depth interviews was to gather individual perspectives, while the focus group discussion was meant to provide a forum as a collective in order to see if other themes emerged. After the in-depth interviews were complete, a focus group discussion was held. Ten field workers participated in the focus group discussion. The other six were unable to participate in the focus group discussion because of other competing commitments. Due to the limited number of community-based fieldworkers who turned up for the focus group discussion, only one focus group could be formed. The study did not use individuals who were subjects of the MAL-ED project itself because the main target group of the present report was the community-based field workers and not other s affiliated with the project.

**Fig. 1:**
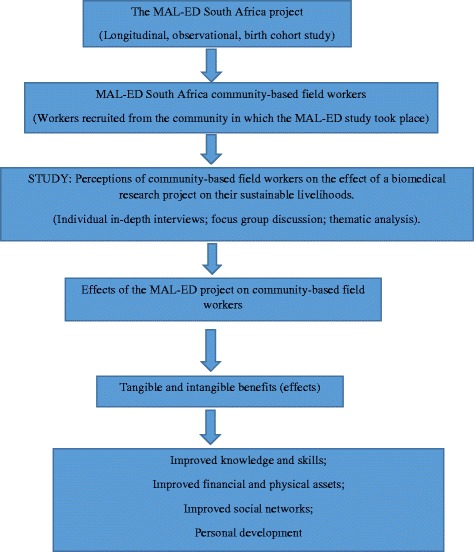
A diagrammatic representation of the conceptual framework of the study. The perspectives of community-based field workers who participated in the MAL-ED project on the effects the project had their sustainable livelihoods were explored. All field workers were eligible for the study, and those from whom consent was obtained participated in the study. The categories of benefits sought (tangible and intangible) of sustainable livelihoods are indicated

In the focus group discussion, one person from the group was chosen to chair the discussion. Another participant served as the scribe. Participants’ responses were written on flip charts once there was consensus among the 10 participants. An investigator of the current report served as the moderator during the discussions. The moderator’s duty was to ensure the smooth flow of the discussion and that participants did not deviate from the topic under discussion. After the completion of the individual interviews and focus group discussion, data were analysed with the Thematic Content Analysis approach [17]. It is part of the inductive approaches derived from the grounded theory. The process involves analysing transcripts, identifying themes from the transcripts and gathering together examples of those themes from the text. The thematic content analysis was ideal for this current study because the study involved the transcribing of data and coming up with themes which matched the data collected. Audio tape recorded responses were transcribed first. All the qualitative data were then captured, coded and analysed using the ATLAS.ti version 7.5.10 software. A diagrammatic representation of the conceptual framework of the study is shown is Fig. 1.

## Results

The findings of the study whose aim was to explore the perceptions of community-based field workers regarding the effects of the MAL-ED South Africa project on their sustainable livelihoods are presented here.

### Demographics of study participants

Twenty-one participants out of the target population of 31 people (16 MAL-ED project field workers and 5 community leaders) participated in the study. The participants’ ages ranged from 23–42 years for project field workers, and 52–67 years for community leaders. Of the 21 participants, nine were women. Four of the nine female participants were married and five of the 12 male participants were also married. Three community-based field workers had post high school diplomas and 13 had high school diplomas as the highest academic qualification. Two community leaders had high school diplomas, while the others did not complete high school education.

### Benefits of the project as perceived by community-based field workers and leaders

Generally, all the community- based field workers expressed positive views on how the MAL-ED South Africa project had improved their livelihoods. They indicated that they had been empowered through accruing enhanced knowledge and skills (human capital development), earning more regular income than before (financial benefits), enhanced socialisation, acquiring physical/material assets and personal development. In addition to this, the field workers expressed the desire to receive training in areas such as public relations, effective communication, problem solving and confidence building. The general observation is that the community-based field workers, irrespective of age or gender, expressed similar views regarding the effects of the study on their sustainable livelihoods. The views of the community leaders on the effects of the study corroborated the views expressed by the individual field workers. All these issues are described in detail in the following sections of the paper.

### Human capital

Most field workers cited acquisition of research skills and knowledge as one of the benefits that accrued to them due to participating in the MAL-ED project. Participants alluded to the fact that they were now aware that conducting a research is not an easy task. They learnt that carrying out a research requires a lot of time, focus, skills and patience. Some of the skills that the participants learnt included filling in forms, obtaining codes for food recipes, weighing and measuring the height of children, collecting biospecimens such as urine and stools, and having a good rapport with the mothers of the children from whom the data were collected. The skills acquired helped some of the participants to subsequently secure jobs. Some of the field workers made mention of the fact that they had gained knowledge about malnutrition and what food to give to their children. Evidence to support this assertion is embedded in the verbatim quotes presented below to illustrate the knowledge and skills that some participants acquired from the project;

*‘Filling in the forms and searching for codes was difficult at first but now I know how to* *do it. I now know how to approach participants, how to weigh children, how to convince* *parents to allow us to collect data.’ (Participant X, 31 years old).*

*‘I did not know about malnutrition and now I have gained some knowledge about it. I know*

*about giving children food that has value and clean water.’ (Participant X, 35 years old).*

Community leaders expressed views similar to those of the fieldworkers when asked about the human capital benefits of the MAL-ED South Africa project to members residing in their community. Some community leaders felt that community members had gained knowledge about giving children healthy food and maintaining healthy life styles. Parents were made aware of the importance of taking children to the hospital if they were unwell. Furthermore, they alluded to the fact that the community field workers had received training on research involving children and also their data collection skills had been enhanced. Also, community leaders were of the opinion that the skills and knowledge gained would help improve the curriculum vitae of the community workers, their livelihoods and those of other community members.

The following are some of the sentiments expressed by the community leaders;

*‘The project teaches parents knowledge about health issues. The project is teaching the* *community how to avoid diseases. There is a change in the community, even young* *children know how to keep healthy.’ (Community leader Y, 63 years old).*

*‘The project helps create jobs for the community. The workers also get training. The* *project is helping CVs of workers.’ (Community leader Y, 66 years old).*

The above sentiments highlight the importance of acquisition of research-specific skills and knowledge as an empowerment for those involved in the project with other community members indirectly benefitting from the project.

### Financial and physical capital

Another observation was that the field workers had reaped financial benefits from the project. The allowances they received from the project were used for their general upkeep, to cover other expenses such as paying for their driving lessons, assisting other family members by giving them pocket money and purchasing physical assets such as refrigerators, stoves, satellite television subscription and microwave ovens. Among the field workers were some who indicated that they used their allowances to further their education. Some used it for much bigger projects such as building a house or extending existing ones. Some of the field workers said the following in support of the claims above:

*‘I got financial help; paid the cost for my driver’s licence with MAL-ED money. The money also helped me to complete my Grade 12. (Participant X, 30 years old).*

*‘I extended my house, bought a bed, DSTV (satellite TV), fridge and* *microwave.’ (Participant X, 31 years old).*

Community leaders corroborated the views of the community-based field workers and stated that the project had helped create jobs for the field workers and that their participation had helped them earn some income which they could use for the upkeep of their families thereby reducing poverty.

The above sentiments highlight the financial and physical benefits that accrued to the community-based field workers because of their involvement in the project. Accumulation of physical assets is one way used to measure one’s wealth, in particular among the people in rural areas. This is crucial for reducing vulnerability to poverty.

### Social capital

Some field workers indicated that the project had assisted them to establish strong social and general interpersonal relationships. They found themselves being able to network, socialise and work as a team better than before they became members of the MAL-ED project. The following are some of the sentiments shared in support of these assertions:

*‘The project helped me to interact and socialise with different people and to work as a* *team.’ (Participant X, 27 years old).*

‘*I like working with colleagues, helping each other to do things. Being with others helps* *relieve stress*. (*Participant X, 32 years old).*

Community leaders concurred with the views expressed by the field workers. They reiterated that the project had helped in building relationships between field workers and community members. Establishing networks is a critical component of social life. It connects persons and enables them to have up to date information on what is happening around them and beyond. Networking also helps open doors for those seeking employment, counselling, get new ideas or insights about social or work related issues. Interacting with people, team work, creating friendships and socialising are crucial ‘ingredients’ for social capital.

### Personal assets/development

The involvement of community-based field workers in the MAL-ED project motivated some of them to pursue further studies. This came as a result of personal and collective reflection on their status, leading to the decision that if they acquired more knowledge and skills they could access and compete for various opportunities such as jobs. Below are some extracts from the participants, which attested to this:

*‘I now want to be a dietician because of the project. I want to help people know about* *healthy foods.’ (Participant X, 35 years old).*

*‘I have grown up in the way of talking and in my personal growth. I now have the urge to*

*pursue education. I want to register for a degree.’ (Participant X, 31 years old).*

The views expressed above show that projects of this nature can rekindle community members’ desire for further education and training. Also evident in what the field workers said was the improvement in one’s character or traits. It was clear that developing and maintaining a positive character helped build and strengthen the relationships the field workers have beyond the project environment. This is crucial because it is an ingredient that solidifies the foundation of strong, cohesive families and communities.

#### Benefits of the MAL-ED project as perceived by the in-depth interviewees, focus groups and community leaders

The results obtained through both the in-depth interviews, focus group discussions and community leaders are presented in Table 1. It was particularly important to combine the findings because the focus group technique and the community leaders’ responses were specifically used to cement what came out of the individual in-depth interviews. A few sentiments that highlighted the empowering nature of the project, derived from in-depth interviews and the focus group are presented per livelihood capital in Table 2.

**Table 1:** Benefits of MAL-ED project as perceived by community-based field workers

	Empowerment	Individual interviewees (field workers)	Focus Group	Community leaders
1.	Knowledge about child growth and malnutrition	We learnt about children’s health and what they should be fed on. (16)	●	●
2.	Acquisition of knowledge various skills	I can now communicate with different people and I now know that I have to wash my hands after every nappy change. (16)	●	●
3.	Knowledge about conducting research and data collection	I now know how to conduct research and how to collect data and fill in forms. (5)	●	●
4.	Acquisition of inter-personal skills	I learnt how to work well with people. (11)	●	X
5.	Received financial benefits	I got money. I used it for my general upkeep. (14)	●	●
6.	Acquired physical assets	I bought a fridge, bed, microwave and a television set. (10)	●	X
7.	Got experience in working with children	I know how to spend time with children.(6)	●	X
8.	Personal development	The project improved my CV. (8)	●	X
9.	Social capital benefits	The project helped me socialise with people. (4)	●	X
10.	Water and sanitation	Knowledge that our drinking water is not clean. (3)	●	●

Perceptions from both individual interviews and focus group were grouped together under the category empowerment. The number against each empowerment statement indicates the number of individual interviewees (field workers) who expressed that perception. A similar perception expressed by the focus group and community leaders to the perceptions expressed by the individual interviewees (field workers) is indicated by a bullet sign. An ‘X’ indicates that the community leader was not in a position to express the perception. It was observed that all the perceptions expressed in the individual interviews were also expressed in the focus group discussion

**Table 2:** Sentiments highlighting the empowering nature of the project on community-based field workers from individual interviews and focus groups

Category of empowerment	Comment
Human capital	‘I did not know about malnutrition and now I have gained some knowledge about it.’ ‘I learnt how to measure the height and weight of children.’
Financial capital	‘I received income. I used it for my personal upkeep.’
Social capital	‘I have made a lot of friends. We now treat each other as relatives.’ ‘The project taught me to socialize with different people.’
Physical capital	‘I built an 8- room house.’ ‘I was able to extend my house and buy furniture, stove, fridge and television set.’
Personal assets	‘My passion for nursing has been enhanced.’ ‘I have grown up in the way of talking and in my personal growth.’

These results cement the observations from the in-depth interviews. The benefits that accrued to the field workers fitted into the financial, human, social and physical capital categories. Also crucial was the enhancement of field workers’ personal character or traits. The perceptions on the benefits derived from the project expressed in the focus group discussion tally with those expressed by the interviews. The community-based leaders expressed five of the ten empowerment statements obtained from the individual field workers.

## Discussion

In this study, it was revealed that various benefits accrued to community-based field workers as a result of their participation in the MAL-ED South Africa project. For example, they acquired research skills and knowledge. They pointed out that they had never participated in a community-based study before, let alone a project of this magnitude. Prior to joining the MAL-ED project, they had basic skills such as reading and writing, basic farming, gardening, sewing and vending. The MAL-ED observational prospective cohort study equipped them with skills such as data collection, team work and communication. Furthermore, they gained considerable knowledge on the growth of children and the types of food to give them, developed a better understanding of malnutrition, infection, and research in general. The knowledge and skills they acquired helped them understand and appreciate the work that they were doing and to perform their duties more effectively. Furthermore, the knowledge and skills positively contributed to their personal growth and development. Although this was not for personal development, the project can be viewed as having developed a pool of paraprofessionals in community-based biomedical research. Application of the knowledge and skills would also indirectly empower the communities in which the field workers reside. This would be realised through the use of the acquired knowledge and skills to invest in other community members’ livelihoods. A past study corroborated this view [18]. The same study highlighted the importance of equipping people with knowledge and skills to enable them to realise their full potential. Furthermore, knowledge and skills are critical for both human and economic development, and increases an individual’s employability.

The sentiments expressed above are consistent with the assertion that community engagement involves generating knowledge from research participants, sharing ideas and also empowering community members so that they become better equipped to deal with the numerous challenges they face in their lives [1]. In the current study, human capital development helped unlock the potential of the field workers and enabled them to gain knowledge and skills they required to improve their livelihoods. The same view led to the conclusion that education, be it formal or in-formal, ‘arms the individual with the skills needed for survival, social interactions as well as contribution to societal development [19]. Similar sentiments were made in a study in the Blantyre District of Malawi [3]. In Malawi, respondents expressed their willingness to participate in research that could benefit them as individuals or those that positively contributed to the development of their communities. These views reveal that effective and beneficial community engagement is that which results in the improvement of the quality of life of research participants and their local areas of residence.

Although the MAL-ED project was not designed to create employment for the community-based field workers, the allowances they received because of their involvement in its activities brought temporary financial relief to them. In South Africa, high levels of unemployment, particularly among the youth, is a major challenge. According to [20], the country’s youth unemployment rate is approximately 36.1% among the15-34 years old group. High school graduates, such as most of the community-based field workers who were involved in the MAL-ED project, were not likely to secure meaningful employment. Thus, getting an opportunity to participate in the MAL-ED project was likely to have been a welcome move for them.

Some field workers revealed that they had used the allowances they received from the project to purchase physical assets, namely stoves, microwave oven, fridges, television sets and furniture. Others used the money to construct houses. There was yet another group of field workers who reported having been enabled to form ‘money saving schemes’ (*stokvels*) with their colleagues. This entailed agreeing on the amount that each member contributed each month., and members would receive the combined sum on a rotary basis. Such a strategy enabled some of the participants to raise enough deposit to purchase the assets that they needed. Stokvels are intervention strategies meant to address the problems of poverty and income insecurity in communities [21]. Furthermore, the authors posit that stokvels serve two functions : to address economic insecurity on one hand and social issues on the other. Economic issues encompass financial capital whilst social issues deal with aspects such as social capital of which relationships and networking are some of them.

Asset accumulation is a crucial weapon used to eliminate poverty, especially among the rural poor. Poor people largely depend on their assets to improve their livelihoods [22] and rely on their accumulated assets as collateral when they apply for loans from micro-finance institutions [21]. In a study conducted in some rural areas of Limpopo Province [23], women revealed that they experienced hurdles in accessing financial resources from banks due to their limited asset base they could use as collateral. This supports the argument that lack of assets among the rural poor reduces their access to credit [22]. It can be concluded that one of the positive, though unplanned attributes of the MAL-ED South Africa project, was field workers’ acquisition of assets, which to some extent transformed their livelihoods and possibly increased their credit worthiness.

Improved social capital was another benefit that community-based field workers accrued through their association with the MAL-ED project. According to [24], social capital refers to ‘networks together with shared norms, values and understandings that facilitate co-operation within or among groups.’ Currently, there is no universal definition of social capital. Thus, in the current study social capital encompassed the creation of friendships, socialisation, team work and networking. It was revealed that the field workers socialised with grassroots community members and mothers of the children they monitored. In addition to this, the field workers reported that they worked as a team, and also networked with people in various locations using their mobile phones or via social media platforms such as *WhatsApp* and *Facebook.* Establishment of ‘money lending’ schemes (*stokvels*) was another major highlight of the growth and development of social capital among the field workers. Through the stokvels, values such as trust, honesty and mutual support were developed and enhanced [21, 25]. Strong social ties were built and strengthened. Social cohesion is fundamental in the development of communities. The pooling together of their labour, resources and the building of networks helps strengthen community ties and enhances community development. Such measures resonate with the spirit of ‘Ubuntu’, a philosophy adopted by South Africa, which puts emphasis on aspects such working as a collective, trust, sharing and caring for one another [26]. Such attributes assist in building and strengthening social cohesion.

Some field workers emphasised that one of the most notable benefits of serving in the MAL-ED project was the realisation of personal development. Increased motivation to pursue further education and training, ability to communicate better, improved confidence to collect data from a range of respondents, heightened self-esteem, and ability to articulate what the project entailed were the most cited personal development attributes. These findings further elevate those obtained in the Intervention with Microfinance for AIDS and Gender Equality (IMAGE) study conducted in Sekhukhune District of Limpopo Province in 2006 [27]. In that study, access to finance was found to have increased the self-confidence of women who had benefitted from small loan funding. The women were said to be better able to participate in decision making processes, in addition to challenging structures that perpetuated traditional gender norms.

### Limitations of the study

The observations of this study should be understood in the context of some limitations. Firstly, two participants, who were asked to leave the study because of incompetence, declined to participate. And four others could not be reached to obtain consent to participate. As a result their perceptions are not known. Secondly, the limited number of participants allowed for only one focus group discussion, and as such it is not certain whether there would have been more diverse views with additional focus groups. Lastly, the observations presented here are derived from individuals, the majority of whom have not been employed previously; therefore the findings should be understood in that context.

## Conclusion

Notwithstanding the study limitations, various benefits accrued to community-based field workers as a result of their involvement in the MAL-ED project. Although the project was a biomedical research study, it significantly enhanced the sustainable livelihoods of the fieldworkers. They acquired skills and knowledge, besides earning income that helped them to accumulate assets; and were able to build social networks and establish connections. Through their frontline project work, the field workers’ personal development and exposure to community-based research improved.

Community-based biomedical investigators should be cognisant of the fact that their projects might have an effect on field workers personally. Some participants reported that they needed further training in public relations, communication, problem solving and confidence building. These statements suggest an unmet need, and imply that investigators could further benefit workers by considering the addition of the training component in strategies that might empower the field workers. Projects that pursue such a trajectory might perform better if the field workers develop a sense of belonging and ‘own’ them.

## Abbreviations

DfID: Department for International Development
IMAGE: Intervention with Microfinance for AIDS and Gender Equality
MAL-ED: The Etiology, Risk Factors and Interactions of Enteric Infections and Malnutrition and the Consequences for Child Health and Development Project
OECD: Organisation for economic co-operation and Development
UK: United Kingdom
